# BGP-15 Protects Mitochondria in Acute, Acetaminophen Overdose Induced Liver Injury

**DOI:** 10.1007/s12253-019-00721-1

**Published:** 2019-11-08

**Authors:** Farkas Sarnyai, Timea Szekerczés, Miklós Csala, Balázs Sümegi, András Szarka, Zsuzsa Schaff, József Mandl

**Affiliations:** 1 Department of Medical Chemistry, Semmelweis University, Budapest, Hungary; 2 2nd Department of Pathology, Semmelweis University, Üllői út 93, Budapest, H-1091 Hungary; 3 Department of Biochemistry and Medical Chemistry University of Pécs, Pécs, Hungary; 4 Department of Applied Biotechnology and Food Science, Laboratory of Biochemistry and Molecular Biology, Budapest University of Technology and Economics, Budapest, Hungary

**Keywords:** Mitochondrium, Acetaminophen, Liver injury, BGP-15, Reduced glutathione, Jun-kinase

## Abstract

Acetaminophen (APAP) induced hepatotoxicity involves activation of c-Jun amino-terminal kinase (JNK), mitochondrial damage and ER stress. BGP-15, a hydroximic acid derivative, has been reported to have hepatoprotective effects in APAP overdose induced liver damage. Effect of BGP-15 was further investigated on mitochondria in APAP-overdose induced acute liver injury in mice. We found that BGP-15 efficiently preserved mitochondrial morphology, and it caused a marked decrease in the number of damaged mitochondria. Attenuation of mitochondrial damage by BGP-15 is supported by immunohistochemistry as the TOMM20 label and the co-localized autophagy markers detected in the livers of APAP-treated mice were markedly reduced upon BGP-15 administration. This effect, along with the observed prevention of JNK activation likely contribute to the mitochondrial protective action of BGP-15.

## Introduction

Acetaminophen (N-acetyl-*p*-aminophenol, APAP) is the most common painkiller, antifebrile and one of the most frequently used drugs in the world [1]. However, APAP is a dose-dependent hepatotoxin, with approximately 50% of all acute liver failure cases in the USA and UK attributed to APAP overdose [2]. The toxicity of APAP is a complicated process and it is not fully understood. Several organelles and molecules have been shown to contribute to APAP toxicity. A series of experimental and clinical data have been published on the pathomechanism of APAP-induced liver injury [3–6]. At a cellular level, it is generally accepted that mitochondrial damage is a key element of the pathology induced by APAP overdose [7, 8], however, endoplasmic reticulum (ER) stress also develops [4]. The mitochondrial injury occurs in at least two steps [3]. Conversion of APAP to N-acetyl-p-benzoquinone imine (NAPQI) is catalyzed mainly by CYP2E1 in hepatocytes [9]. NAPQI causes oxidative stress through increased ROS generation and adduct formation with proteins and glutathione. The formation of mitochondrial protein adducts due to APAP overdose induced NAPQI formation plays a crucial role in the initiation of APAP induced liver injury [8, 10, 11]. Formation of the aforementioned protein adducts is involved in the inhibition of mitochondrial respiration [12], the subsequent formation of ROS [13] and peroxynitrite in mitochondria [14]. Furthermore, the role of the initial increase in ROS formation upon NAPQI synthesis stimulates intracellular signaling processes [11]. Oxidative stress triggers MAP kinase cascades that lead to phosphorylation and activation of c-Jun amino-terminal kinase (JNK), which in turn is connected to the initial mitochondrial dysfunction. Activation and mitochondrial translocation of JNK in the liver has also been shown to play a central role in the pathomechanism of APAP toxicity [15]. JNK amplifies the already existing mitochondrial oxidant stress and largely contributes to cell death by stimulating MOMP (mitochondrial outer membrane permeabilization) and MPT (mitochondrial permeability transition) [16], which leads to the collapse of mitochondrial membrane potential and to the release of various proapoptotic mediators. Nuclear translocation of AIF (apoptosis-inducing factor) and endonuclease G from the mitochondria is a well-known event of caspase-independent apoptosis, and it was indeed demonstrated in livers of APAP-treated animals [5, 17].

BGP-15 is a hydroximic acid derivative. Its various experimental effects have been demonstrated in a series of different animal models and also in cell cultures. These are protective effects influencing among others heart, skeletal muscle, liver, oocytes, skin and show the involvement of mitochondria [5, 18–24]. Hindrance of ROS elevation and also moderation in JNK activation by BGP-15 have been shown in different experimental models [18]. Phase II clinical observations suggest its antidiabetic effect [25].

We have published protective effects by BGP-15 in acute APAP induced liver injury; it largely counteracted MOMP [5]. In addition, mitochondrial dysfunction and even ER stress were also affected by BGP-15 in different experimental systems [19, 22, 23]. Therefore, the effect of BGP-15 was further investigated focusing mainly on morphological signs of the involvement of mitochondria and on JNK activation. Protective mitochondrial effects of BGP-15 in APAP overdose induced liver injury have been shown on various pathomorphological phenomena, and JNK activation.

## Materials and Methods

In this report, in vivo hepatoprotective effects of BGP-15 in APAP-induced liver injury are shown in mice. The animals (male NMRI BR SPF mice of 25–30 g body weight) were starved for 18 h prior to the administration of a single sub-lethal dose of APAP (450 mg/kg bw, i.p.). APAP was added with or without 100 mg/kg body weight BGP-15; the controls received vehicle or BGP-15 only. The mice were sacrificed after 6 h, blood samples were withdrawn and the livers were dissected (for Western blot, RT-PCR, electron microscopy, immunohistochemistry and metabolic analysis). These studies were conducted in accordance with the laws and regulations of governing authorities, and they were approved by the Epidemiology and Animal Protection Division of the Governmental Directorate of Food Chain Safety and Animal Health.

### Histology, Immunohistochemistry

Samples from mice livers were fixed in formalin and embedded in paraffin (FFPE). 3–4 μm thick sections were prepared and stained by hematoxylin-eosin (H&E).

### Immunohistochemistry (IH)

FFPE sections were used after deparaffinization and endogenous peroxidase blocking applying 1% hydrogen peroxide. For retrieving antigens Target Retrieval Solution (DAKO, Glostrup, Denmark) was used for 30 min in microwave oven, followed by incubation with the primary antibodies against TOMM20 (mAb, 1:200, Santa Cruz Biotechnology Inc., CA, USA), JNK (Phospho-SAPK/JNK, Thr183/Tyr185, 81E11, rabbit mAb, 1:100, Cell Signaling Technology Inc., Leiden, The Netherlands), Beclin1/ATG6 (1:200, Novus Biologicals Europe, Cambridge, UK), LC3 (1:200, polyclonal rabbit NB-100-2331 Novus Biologicals Europe) and p62 (1:1000, monoclonal mouse ab56416, Abcam, Cambridge, MA, USA).

The reactions were carried out with Ventana Benchmark XT automated immunohistochemical staining system (Ventana Medical System Inc., Tucson, AZ, USA) with HRP Multimer based, biotin-free detection technique according to the protocol provided by the manufacturer. Reagents and secondary antibodies were obtained from Ventana and the reactions were visualized by UltraView™ Universal DAB Detection Kit (Ventana). Nuclear staining was performed using hematoxylin. For negative control, primary antibodies were substituted with Antibody diluent (Ventana).

### Electron Microscopy

Samples from liver were fixed in 2.5% glutaraldehyde, followed by 1% OsO4. After dehydration in graded ethanol, the samples were embedded into epoxy resin. Semithick sections were used for the selection of the proper areas, followed by ultrathin sectioning. The uranyl acetate and lead citrate contrasted sections were analyzed and photographed in a JEM 1011 electron microscope (JEOL).

### Sample Preparation and Western Blot Analysis

Approximately 1 mg of the liver was homogenized with Elvehjem tissue grinder in lysis buffer. The lysis buffer contained 0.1% SDS, 5 mM EDTA, 150 mM NaCl, 50 mM Tris, 1% Tween 20, 1 mM Na3VO4, 1 mM PMSF, 10 mM benzamidine, 20 mM NaF, 1 mM pNPP and protease inhibitor cocktail. The lysates were centrifuged with benchtop centrifuge (10 min, 10.000 rpm, 4 °C). Protein concentration of the supernatant was measured with Pierce BCA Protein Kit Assay (Thermo Scientific), and then stored on −20 °C until use.

Samples (50 mg protein) were electrophoresed on 10% SDS polyacrylamide gels and transferred to PVDF membrane (Millipore). Primary and secondary antibodies were applied overnight at 4 °C and for 1 h at room temperature, respectively. Equal protein loading was validated by detection of GAPDH as a constitutively expressed reference protein. Mouse monoclonal anti-GAPDH (Santa Cruz, sc-32,233) antibody was used at 1:20,000 dilution. Primary antibodies: rabbit anti-phospho-SAPK/JNK (THR183/Tyr185) antibody (#9251S), anti-SAPK/JNK antibody (#9252S). Secondary antibodies: Horseradish peroxidase (HRP)-conjugated goat anti-rabbit IgG-HRP (#7074) from Cell Signaling and donkey anti-goat IgG-HRP (sc-2020) from Santa Cruz. HRP was detected with chemiluminescence using Western Lightning Plus-ECL (Perkin Elmer).

## Results

### Effect of BGP-15 Administration on APAP- Induced Liver Injury Seen by Light and Electron Microscopy and Immunohistochemistry

**Fig. 1:**
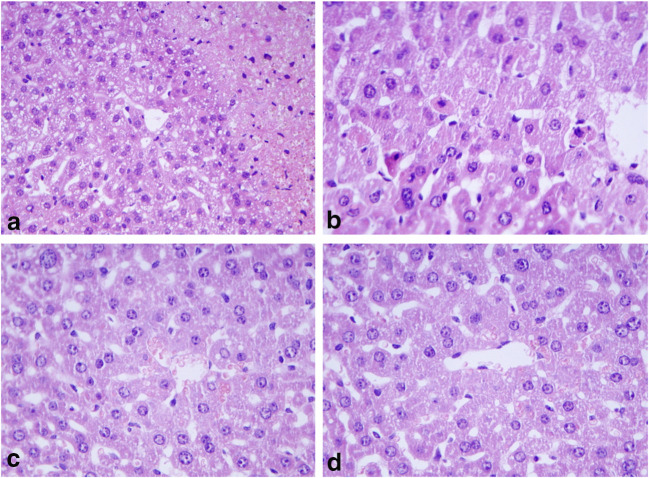
Histology of mouse livers 6 h after treated with APAP (**a**), with BGP15 (**b**), untreated control (**c**) and APAP+BGP15-treatment (**d**). Severe cellular injury in APAP-treated livers, while no alterations in the other samples (formalin fixation, paraffin embedding, hematoxylin and eosin staining). (a, c, d: 150X, b: 250X)

**Fig. 2:**
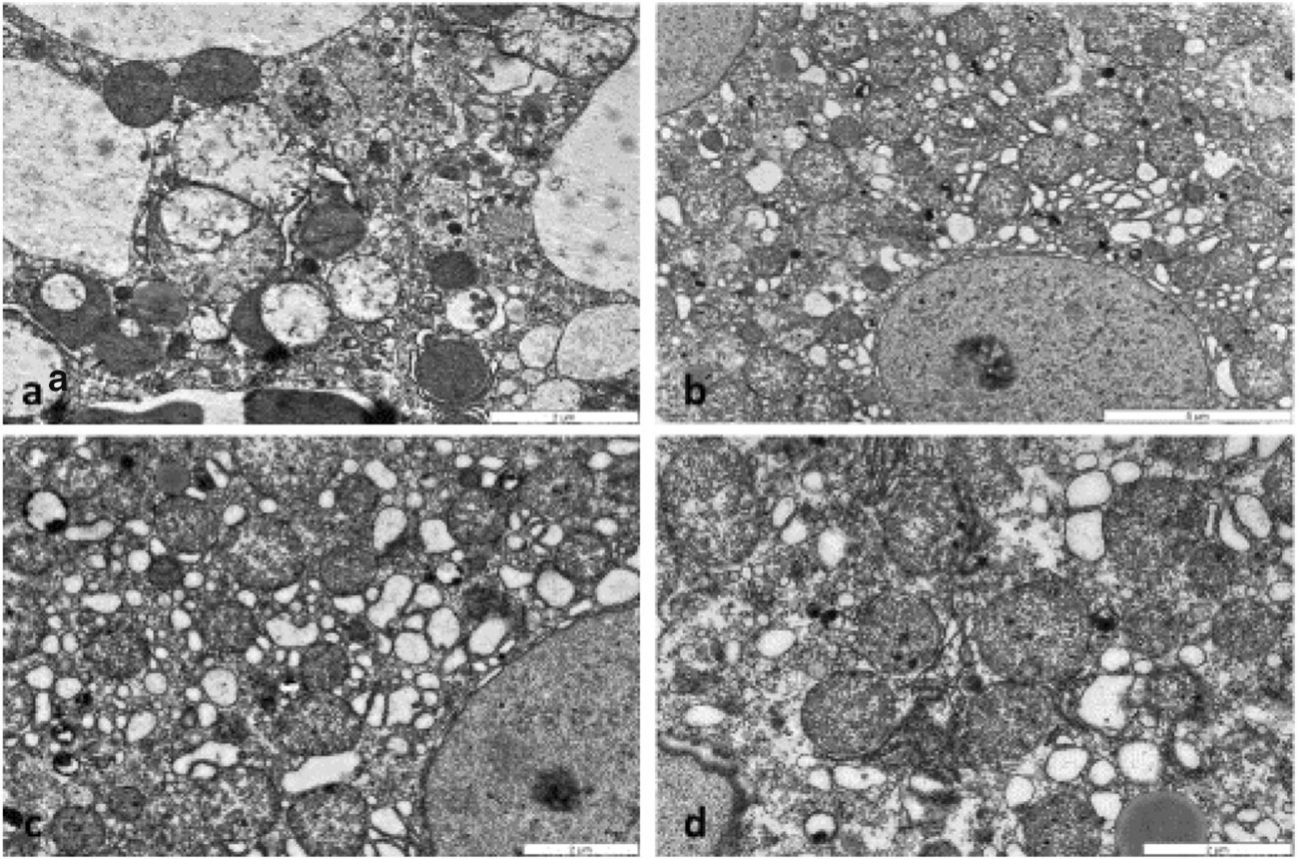
Electron micrographs of mouse livers treated with APAP (**a**), BGP15 (**b**), untreated control (**c**) and APAP+BGP15 (**d**) for 6 h. Severely degraded organelles, some mitochondria lost their cristae, mitophagy in a damaged hepatocyte after APAP-treatment (**a**). Hepatocyte with large number of mitochondria with normal ultrastructure in BGP15-treated (**b**), control (**c**) and APAP+BGP15-treated liver (**d**). (Glutaradehyde+OsO4 fixation, resin embedding)

**Fig. 3:**
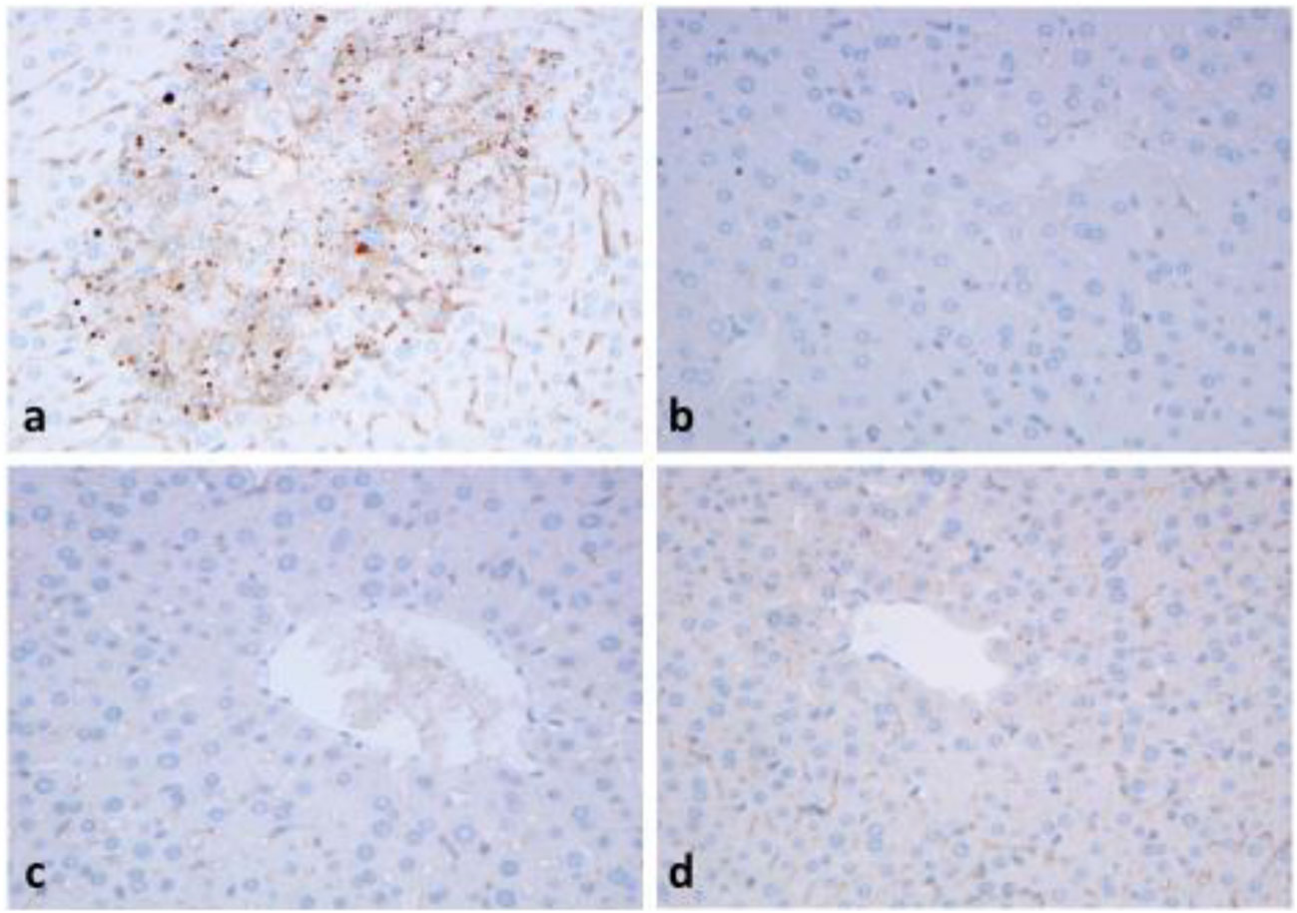
Immunohistochemical reaction with TOMM20 antibody. Mice were treated with APAP (450 mg/kg) and BGP (100 mg/kg) alone, or in combination for 6 h. Intensive granular reaction is detected in the hepatocytes after APAP-treatment in the centrolobular area (**a**). No reaction in the BGP15-treated (**b**) and control livers (**c**) and few granules can be seen after APAP+BGP15 treatment (**d**). (formalin fixation, paraffin embedding, hematoxílin-staining) (150X)

After 6 h of APAP administration, severe liver cell injury, mainly in centrolobular localization was observed (Fig.1a). In contrast, no sign of liver injury as necrosis or apoptosis was present in the BGP15-treated (Fig.1b) or in the control (Fig. 1c) and APAP+BGP16-treated (Fig.1d) samples. The severe cellular injury was well presented by electron microscopy (Fig. 2). Distorted, vacuolated mitochondria were observed, many with lost cristae (Fig. 2a), some of them localized in lysosomes. The BGP-15-treated (Fig. 2b), control (Fig. 2c) and APAP+BGP-15-treated (Fig.2d) hepatocytes demonstrated normal mitochondrial morphology. Immunohistochemical reactions using anti-TOMM20 antibody, demonstrated large numbers of positively-stained brown granules with variable sizes in the injured hepatocytes after APAP-administration (Fig. 3a), mainly located in the centrolobular zones, while the BGP-15-treated (Fig. 3b) livers were more like those of the control animals (Fig. 3c) and only very few brown granules were observed in the APAP+BGP15-treated livers (Fig. 3d).

**Fig. 4:**
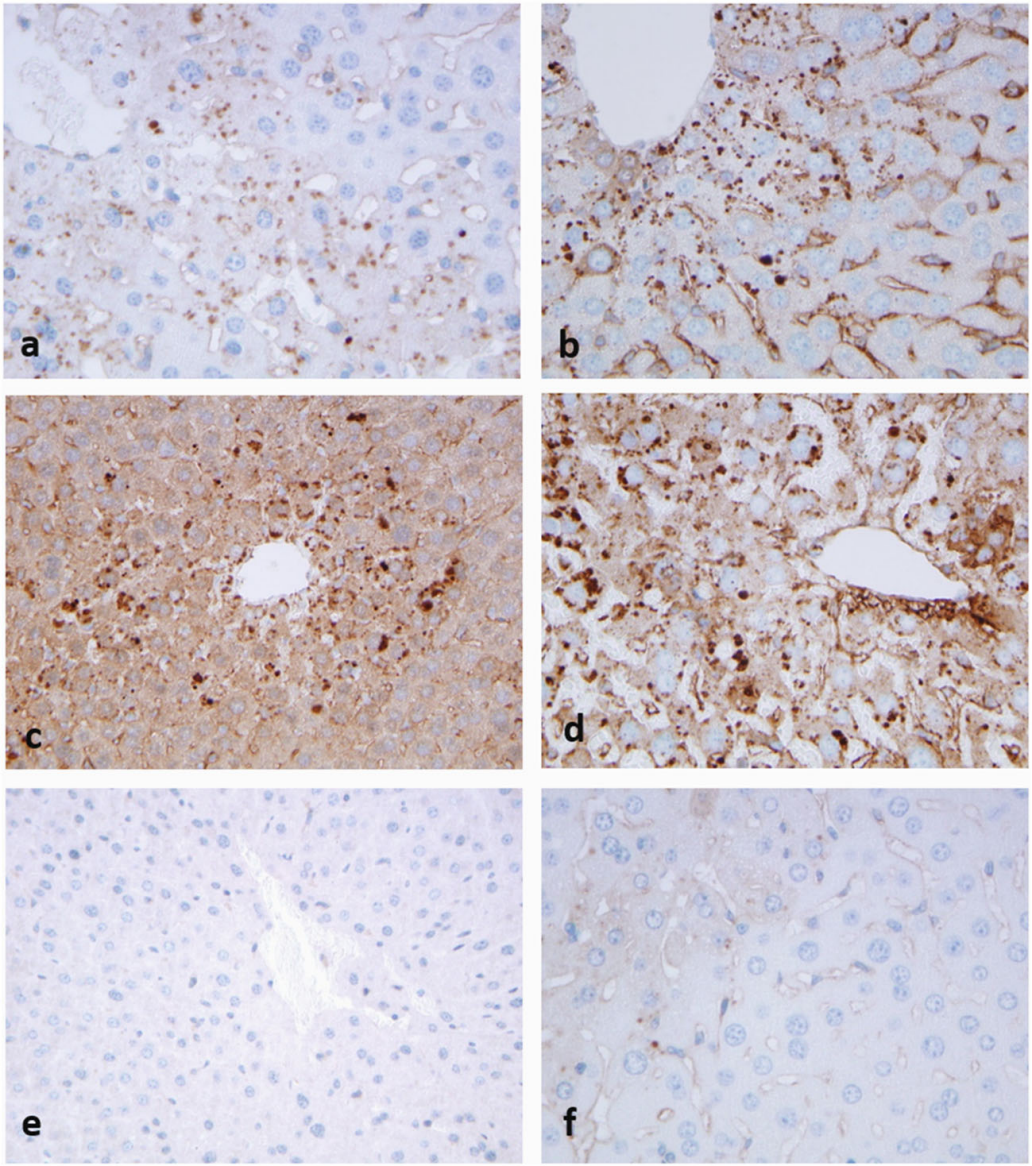
Strong granular immunohistochemical reaction with antibodies to TOMM20 (**a**), beclin1 (**b**), LC3 (**c**) and p62 (**d**) after APAP-treatment. No reaction in the control, untreated livers (**e**) and few granules can be seen after APAP+BGP15 treatment (**f**). (formalin fixation, paraffin embedding, hematoxylin-staining). (250X)

Parallel FFPE sections were used to detect autophagy markers in APAP-treated livers as compared to TOMM20 (Fig. 4a). Immunohistochemical reaction to autophagy markers beclin1 (Fig. 4b), LC3 (Fig. 4c) and p62 (Fig. 4d) presented granular positive reaction in the hepatocytes, in similar centrolobular localization as TOMM20. In contrast no positive reactions could be detected in the control livers (Fig. 4e), and only few granules could be seen in the APAP+BGP15-treated hepatocytes (Fig. 4f).

### Effect of BGP-15 Administration on JNK Activation

**Fig. 5:**
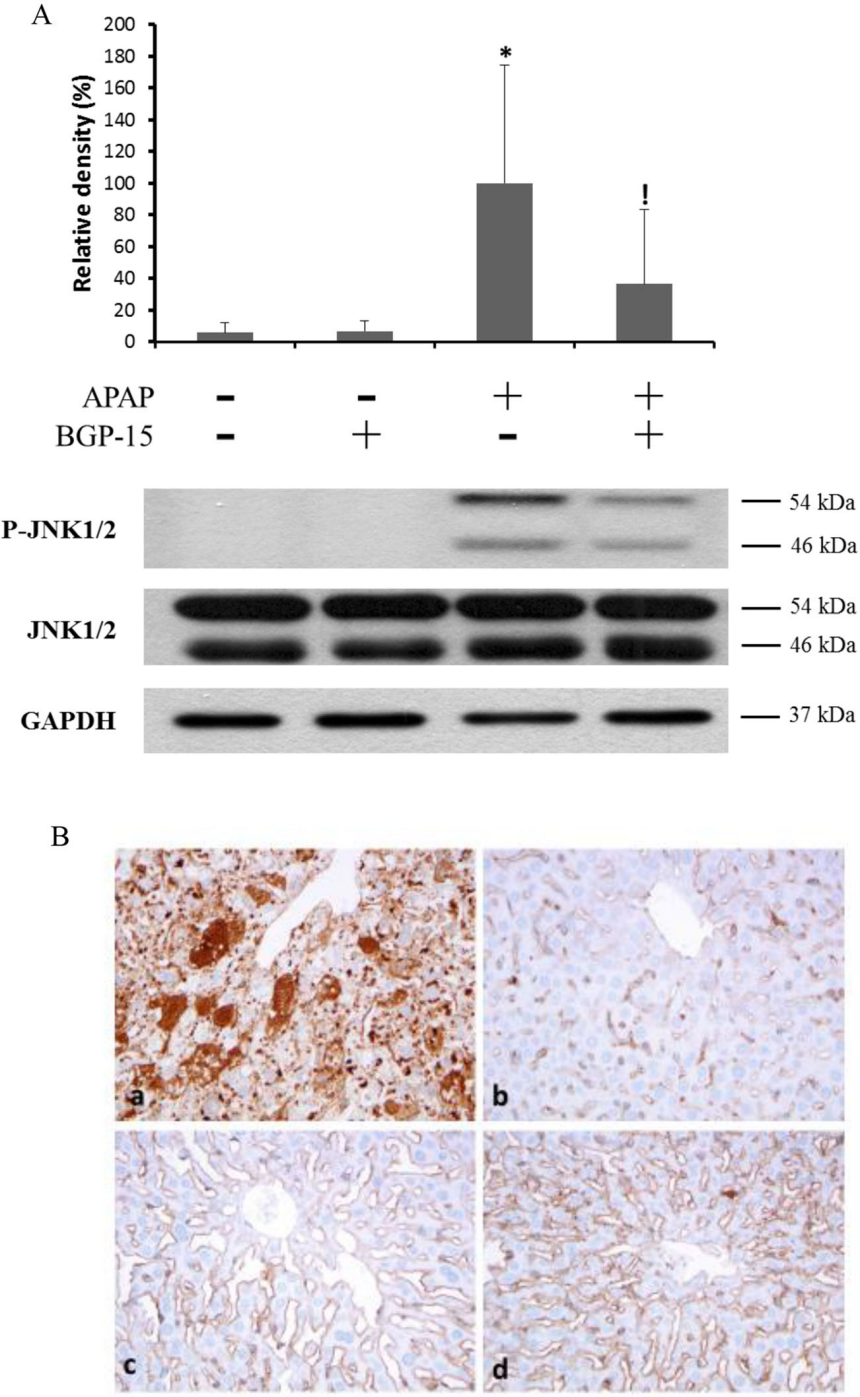
Effect of BGP-15 on APAP-induced JNK phosphorylation in mouse liver. A. Western blot using specific antibodies as indicated, protein lysate from liver tissues. Mice were treated i.p. with APAP (450 mg/kg) and BGP (100 mg/kg) alone, or in combination for 6 h. Liver homogenates were prepared and phosphorylation and expression levels of c-Jun N-terminal kinase (JNK) were investigated by western blot analysis by using specific antibodies against phosphorylated (upper panel) and total (middle panel) JNK. The picture shows typical blot images obtained in one of the four independent experiments, each including three parallels. The results were quantified by densitometry, normalized to GAPDH (lower panel) and expressed as relative band densities in the percentage of APAP-treated. Data are shown as mean values ± S.D.; *n* = 7–12; *P < 0.005, v.s. untreated control;^!^*P* < 0.005, v.s. APAP-treated. B. Immunohistochemistry with antibody against p-JNK. Severely injured hepatocytes are highlighted by dark brown stained areas after APAP-treatment (**a**) as compared to the BGP15-treated (**b**) and untreated control (**c**). After APAP+BGP15- treatment only few brown granules can be seen (arrow) (**d**). (formalin fixation, paraffin embedding, hematoxylin staining,) (150X)

The level of JNK phosphorylation as an indicator of the activation of stress kinases was investigated in the liver samples by western blot analysis (Fig. 5a). Consistent with previous observations, a dramatic, nearly 20-fold increase in phosphorylated JNK level was found in the livers of mice after a sub-lethal APAP treatment. BGP-15 alone did not cause any significant change in JNK phosphorylation, however, co-treatment with BGP-15 remarkably reduced the JNK activating effect of APAP in the mouse liver, i.e. the increase in phosphorylation was only 6-fold in the BGP-15 co-treated animals (Fig. 5a).

After APAP administration, pJNK immunohistochemical reaction was strongly positive (Fig. 5a.), as compared with the BGP15-treated (Fig.5b) and untreated control (Fig. 5c). APAP+BGP-15 treatment resulted in a decreased reaction, only few granules of various sizes were detected in the hepatocytes (Fig. 5d).

## Discussion

Our data presented in this paper further support the protective mitochondrial effects of BGP-15 in acute APAP overdose induced liver injury, which was demonstrated in our previous paper [5]. BGP-15 greatly improves mitochondrial morphology and causes a marked decrease in the number of damaged mitochondria. The attenuation of mitochondrial damage by BGP-15 is further demonstrated by immunohistochemical methods. These changes are accompanied by the parallel increase or decrease of autophagy markers.

Originally, BGP-15 was introduced as an inducer of heat shock proteins (hsp) [26] and various recently published BGP-15 effects were also coupled to hsp induction [18, 20]. These BGP-15 effects make the drug potentially effective in influencing also endoplasmic reticulum (ER) stress related phenomena [22]. However, several effects of BGP-15 can be hardly explained solely by its hsp inducer actions [21].

The presented findings highlight the improvement of mitochondrial functions as a mechanism underlying the beneficial effects of BGP-15. As a result of the oxidative stress, JNK 1 and 2 are activated [27]. Then the phosphorylated (activated) JNK translocates to the mitochondrial membrane and generates further mitochondrial oxidative stress that triggers the opening of MPT pore, which can finally lead to cell death [28, 29].

Observations published recently suggest that BGP-15 affects mitochondrial fusion-fission cycle, preventing mitochondrial fragmentation [23]. In accordance with these observations, BGP-15 administration results in a decrease in the number of damaged mitochondria as it has been demonstrated in our recent study, which is accompanied with the increase in mitophagy. The central role of mitochondria in apoptosis has long been known, and the participation of mitochondrial pro-apoptotic factors in ER-derived apoptosis has also been revealed. The increased number of apoptotic cells without a simultaneous activation of effector caspases highlights the role of caspase-independent mechanisms in APAP-induced acute hepatotoxicity [30].

Mitochondrial dysfunction and autophagy are involved in a number of acute and chronic liver diseases, as drug induced liver injury, fatty liver or hepatocellular carcinoma [31]. BGP-15 has been shown to affect various model liver injuries, as acetaminophen overdose induced liver damage and tumour formation in murine hepatoma xenografts [5, 32, 33]. Our recent data further confirm mitoprotective mechanism of BGP-15 action in an accordance with parallel changes in mitophagy in acetaminophen overdose induced liver injury.
